# A complication of an axillary intra-aortic balloon pump

**DOI:** 10.1093/jscr/rjae092

**Published:** 2024-03-05

**Authors:** Eileen Kodack, Riya Patel, Carain Bonner, Enrique Pantin

**Affiliations:** Department of Anesthesiology and Perioperative Medicine, Robert Wood Johnson Medical School, New Brunswick, NJ 08901, United States; Robert Wood Johnson Medical School, Rutgers University, New Brunswick, NJ 08901, United States; Department of Anesthesiology and Perioperative Medicine, Robert Wood Johnson Medical School, New Brunswick, NJ 08901, United States; Department of Anesthesiology and Perioperative Medicine, Robert Wood Johnson Medical School, New Brunswick, NJ 08901, United States

**Keywords:** intra-aortic balloon pump (IABP), intra-aortic balloon pump complication, intra-aortic balloon pump malposition, ascending aorta mass, descending aorta, transesophageal echo

## Abstract

Intra-aortic balloon pumps (IABPs) are used to mechanically temporize a failing heart by decreasing afterload while increasing coronary perfusion pressure of the heart while more definitive treatment is sought. We report a case of a 65-year-old male with nonischemic cardiomyopathy, atrial fibrillation, thyroiditis, and non-Hodgkin lymphoma who presented with worsening heart failure. He underwent a percutaneous placement of a left axillary IABP with seemingly no complications. Approximately 3 weeks post-placement, the patient was taken for a heart transplant when an intraoperative transesophageal echo showed that the IABP was in the aortic arch and ascending aorta, instead of its proper placement in the descending aorta. The patient’s arterial line showed waveforms appropriate for an IABP patient, and the patient showed no signs indicative of improper placement. This erroneous placement carried the potential to affect the aortic valve function, injure the aortic intima and/or occlude the aortic arch vessels. .

## Introduction

An intra-aortic balloon pump (IABP) is a device for patients with low cardiac output and/or used to decrease afterload while increasing coronary perfusion pressure [1, 2]. This device is inserted into the common femoral artery or via the axillary artery with the IABP residing in the descending aorta and inflating during diastole while deflating during systole [3]. It increases intra-aortic pressure during diastole to improve coronary perfusion and lowers aortic pressures during left ventricular ejection to enable favorable hemodynamics [4]. We present a case that demonstrates that a left axillary IABP can migrate from the descending aorta into the ascending aorta and arch and can function appropriately with no apparent signs of misplacement, or was inadvertently malpositioned since its insertion as intraoperative fluoroscopic images were not available.

## Case report

A 65-year-old male with a history of cardiomyopathy with a left ventricular ejection fraction of 20%–25% on home milrinone, cardiac resynchronization therapy, bicuspid aortic valve status post aortic valve replacement in 2002, anti-cardiolipin antibody positive, and non-Hodgkin’s lymphoma presented to the emergency room with worsening heart failure. He had 22 s of ventricular tachycardia (VT) resistant to anti-tachycardic pacing and received one effective synchronous shock.

**Figure 1:**
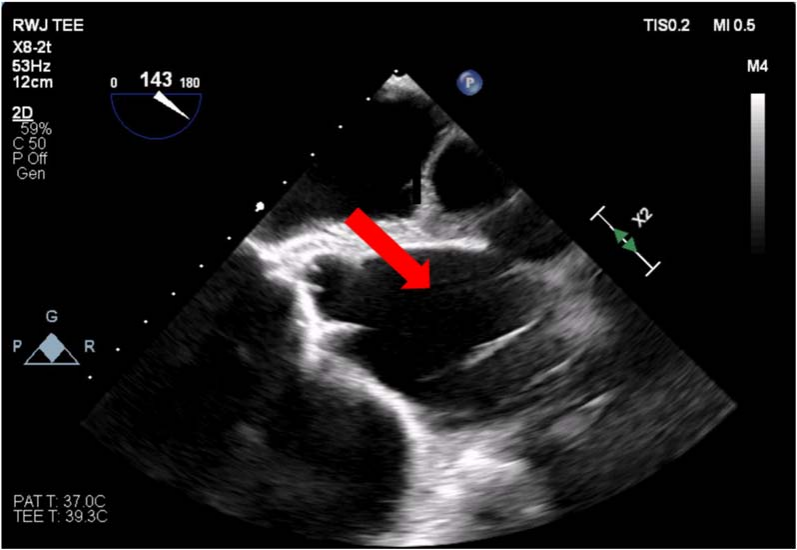
Transesophageal image of the aortic root and ascending aorta with IABP present.

**Figure 2:**
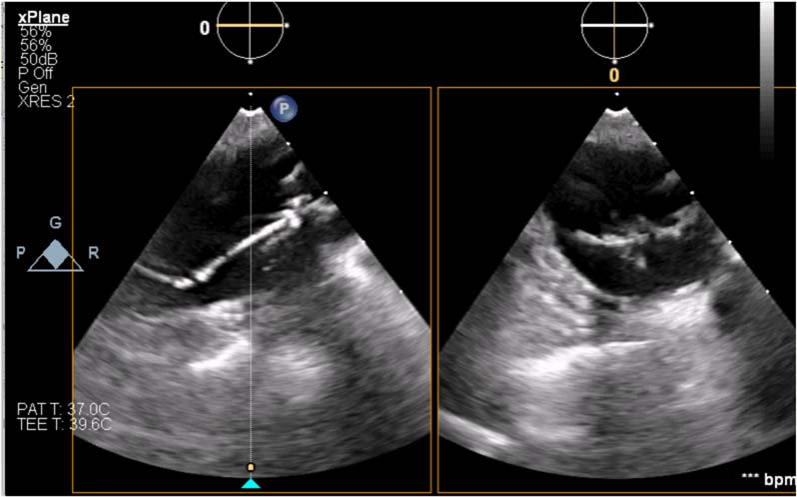
Transesophageal image of the ascending and descending aorta with mispositioned IABP present.

The patient’s milrinone dose was unable to be increased due to his risk for VT and his marginal blood pressures. A 50 cc 8 French left axillary IABP with proximal and distal markers was placed under fluoroscopy and transesophageal echo (TEE) guidance to prevent worsening of end-organ dysfunction and cardiogenic shock. About 3.5 weeks later, the patient received a heart transplant. His 20 gauge right radial arterial catheter was used for monitoring. There were no identifiable changes in the arterial line and the pressure waveforms. The patient was ambulatory in the intensive care unit (ICU) and showed no signs of decreased perfusion. After induction of general anesthesia, TEE showed the IABP in his aortic arch and ascending aorta with its tip placed proximally in the descending aorta (Figs 1 and 2).

## Discussion

This highlights one complication of an axillary-placed IABP: inadvertent mispositioning in the ascending aorta. IABPs are supportive devices that promote forward flow, improve perfusion, and decrease afterload [5]. Most IABPs have a balloon catheter with radiopaque markers at each end indicating where the IABP is in the descending aorta [4]. Axillary-placed IABPs must have a marker at each end of the balloon, as having only one marker could make it difficult to locate the distal marker within the mid-abdominal aorta [4].

When the IABP is placed via the common femoral artery, the marker should be 1 to 2 cm distal to the left subclavian artery or 2 cm above the carina on the chest X-ray (CXR) [1]. This position enables maximum augmentation of coronary blood flow and minimizes the risks of embolization into a cerebral vessel and occlusion of the left subclavian artery [6]. If the IABP is placed via the axillary or subclavian artery under fluoroscopy, the ideal level of the proximal marker of the balloon is at the left main bronchus and the ideal level of the distal marker is at the mid-abdominal aorta. Proper IABP positioning from the common femoral and axillary arteries is essential to prevent complications [7].

Mobility of patients with axillary IABPs may predispose patients to increased risk of mispositioning compared to those placed in the femoral artery [8]. For example, obstruction of the subclavian artery can lead to unequal or absent radial pulses and/or dampening or loss of arterial waveform in the ipsilateral radial artery. Additionally, carotid artery obstruction may lead to changes in consciousness. Furthermore, axillary-placed IABPs are more prone to vascular complications (e.g. aortic dissection and aortic rupture), folding of the balloon in the aortic arch and/or ascending aorta, left upper extremity ischemia, pseudoaneurysm of the axillary artery, and cerebrovascular accidents [9, 10]. As the IABP can fold upon itself, the balloon can potentially cross the aortic valve and even enter the left ventricle.

**Figure 3:**
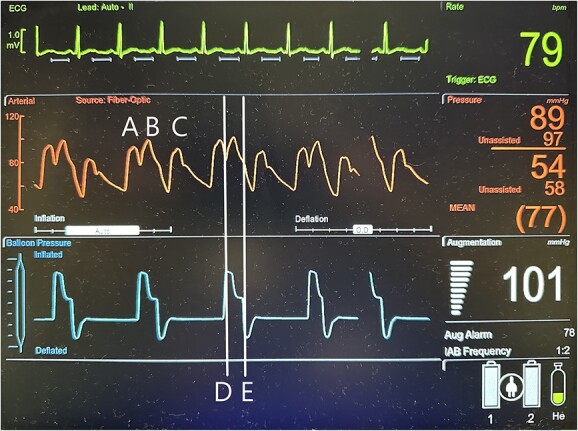
Illustration of a normal IABP arterial waveform with IABP augmentation of diastolic and systolic pressures and normal IABP pressure waveform corresponding to inflation and deflation of the device with a distinctive pressure plateau. A = unassisted heart beat arterial pressure waveform; B = IABP generated arterial pressure waveform; C = assisted heat beat arterial pressure waveform; D = IABP balloon inflation pressure waveform; E = IABP balloon deflation pressure waveform.

Additionally, augmentation of the IABP can be monitored by arterial line and pressure waveform tracings. For optimal effect, the IABP must be properly timed with the cardiac cycle. When timing IABP inflation and deflation by the patient’s electrocardiogram (EKG), the balloon inflates at the beginning of diastole, which corresponds with the middle of the T-wave on the EKG, and deflates at the end of diastole, which corresponds with the R-wave on EKG [11]. A normal functioning IABP should produce a distinct arterial waveform with diastolic augmentation, assisted systole, assisted end-diastolic pressure, and a distinct balloon pressure waveform (Fig 3) [11, 12].

**Figure 4:**
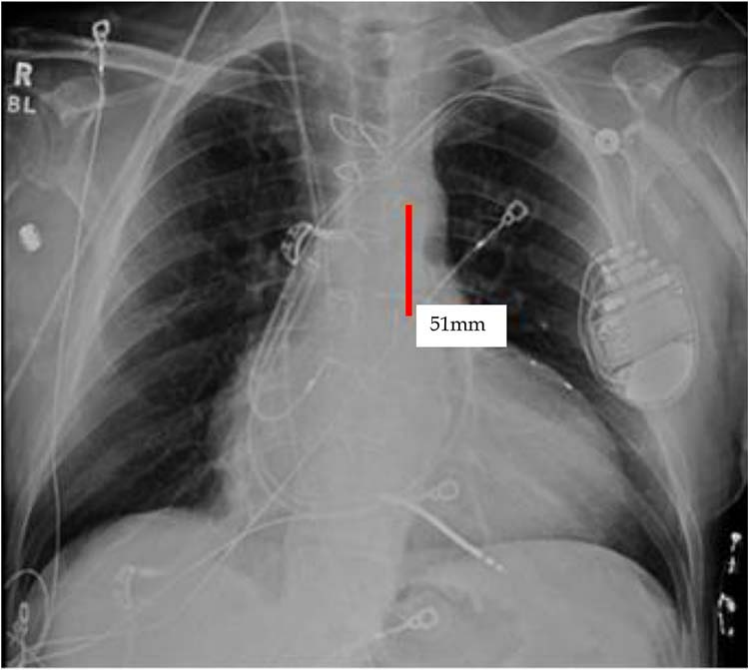
Initial CXR: note the proximal and distal radiopaque markers of the IABP are visualized a few centimeters (5.1 cm) from each other; the proximity of the two markers indicates that along the path of the IABP, it has folded over itself causing the two markers to be abnormally close, as the 8Fr. balloon is 258 mm long, thus the markers should be separated by that much.

TEE guidance and fluoroscopy were used during the insertion of this patient’s left axillary IABP. The TEE note post-IABP insertion states that ‘the balloon is seen from the distal end of the aortic arch extending into the descending aorta.’ A same-day post-insertion CXR in the ICU showed a shortened IABP at about 5.1 cm long, indicating that the balloon must have been folded over itself to allow both markers to be so close to each other (Fig. 4).

**Figure 5:**
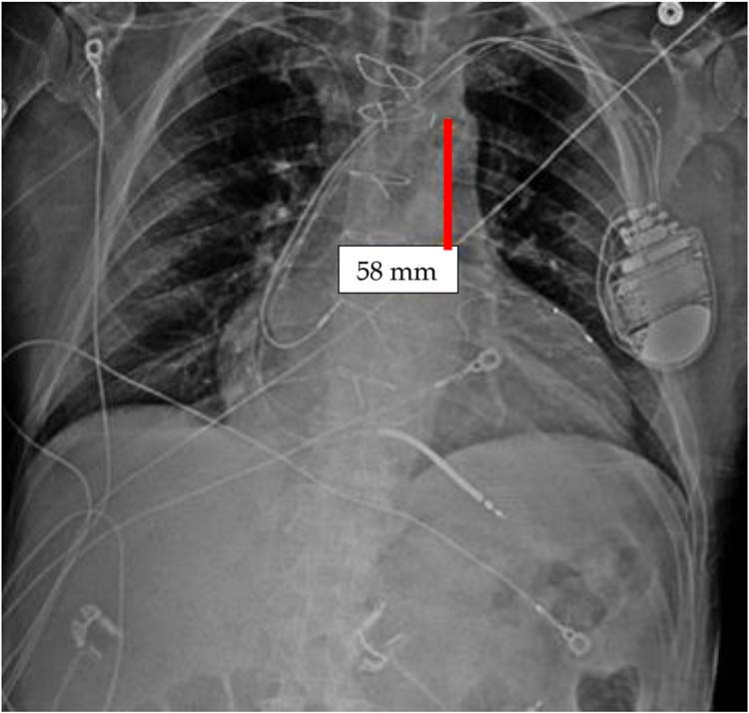
CXR done 21 days later, which shows the markers still not properly placed but the distance is about the same (5.8 centimeters) as the original position; additionally, the shadow of the inflated helium balloon can be seen in the ascending and aortic arch.

It appears that the IABP became mispositioned after its initial placement as seen on follow-up CXR (Fig. 4). Despite almost daily CXRs, this IABP mispositioning was missed by the managing team and radiologists, probably due to interference from the automatic implantable cardioverter defibrillator (AICD) leads, EKG leads, and sternal wires. Twenty-one days later (Fig. 5), the radiologist erroneously read that the IABP had been removed, so CXRs were never repeated. Figure 5 shows that the IABP was still folded over itself but now with a shadow in the arch/ascending aorta from the migrated IABP.

Drexler et al. describes a similar case in which a 73-year-old male had a left axillary IABP placed as a bridge to a left ventricular assist device. The IABP was initially properly placed, but the next day, a CXR showed that the IABP had folded on itself in the ascending aorta [13].

TEE can be used to diagnose mispositioning of IABPs. IABP markers are difficult to visualize, and the patient may not develop symptoms that indicate IABP migration and necessary repositioning. This case highlights the importance of reviewing images ourselves, as the IABP position can change or be misplaced without any symptoms.
